# Risk Analysis by Age on the Burden of Meningococcal Disease in Spain

**DOI:** 10.3390/vaccines10040592

**Published:** 2022-04-12

**Authors:** Irene Rivero-Calle, Peter Francis Raguindin, Jacobo Pardo-Seco, Federico Martinon-Torres

**Affiliations:** 1 Translational Pediatrics and Infectious Diseases, Department of Pediatrics, Hospital Clínico Universitario de Santiago de Compostela, 15706 Galicia, Spain; irina667@hotmail.com; 2 Genetics, Vaccines and Pediatric Infectious Diseases Research Group (GENVIP), Hospital Clínico Universitario and Universidad de Santiago de Compostela (USC), 15706 Galicia, Spain; j.pardoseco@gmail.com; 3 CIBER Respiratory Diseases (CIBERES), Institute of Health Carlos III, 28029 Madrid, Spain; 4 Institute of Social and Preventive Medicine, University of Bern, 3012 Bern, Switzerland; peter.raguindin@ispm.unibe.ch; 5 Swiss Paraplegic Research, 6207 Nottwil, Switzerland

**Keywords:** meningococcal vaccines, meningococcal infections, invasive diseases, *Neisseria meningitides*

## Abstract

We conducted an age-based risk analysis of meningococcal disease in Spain to provide prospects on a rational vaccine schedule in pediatrics. We used the National Hospital Registry to estimate meningococcal hospitalization rate. Population census for each year was used as the denominator in computing the hospitalization rate. We computed the odds ratio of each age using <1 year old as a reference group. From 1998 to 2017, 13,554 hospitalized cases were diagnosed, with a declining trend across the years. Infants (<1 year, *n* = 2425) and children (1–14 years, *n* = 6053) comprised the majority of all hospitalized meningococcal disease in Spain (62.5% or 8474/13,554). The incidence of hospitalization decreased dramatically with age from 56.2/100,000 in <1-year-old children to 1.3/100,000 in >5-year-old children. There was a dramatic decline in risk in 1 year (OR 0.58) to 4 years of age (OR 0.21). The risk continued to decline until 13 years old. Afterward, it had a minimal upward trajectory observed at 14–17 years old (OR 0.08). Infants and adolescents are at continued risk of invasive meningococcal disease in Spain. The highest risk occurs in infants. Surveillance data, together with evidence on long-term immunogenicity and capacity for herd effect, should be considered for a more relevant immunization schedule.

## 1. Introduction

Recommendations on the schedule of routine immunization programs have been based on multiple factors, including product safety and effectiveness, economic assessment, and local epidemiology of the disease [1,2,3]. In Spain, meningococcal C vaccines were first recommended in the routine infant immunization program starting in 2000, as a response to a spike in the incidence in 1996–1997 [4,5,6]. The vaccine was initially given at a three-dose primary infant series. Five years later (in 2005), the recommendations were changed into two primary doses and the addition of a booster dose at 1 year of age, as a response to newer evidence of declining immunity documented from the older schedule [4]. Eight years later, in 2013, the recommendations were further revised to add a booster dose at 12 years of age, as newer clinical trials demonstrated the waning antibody titers in adolescents. This latest approach recognizes the need to improve herd immunity among the broader population [7].

The frequent recommendation changes were a response to newer data on the antibody kinetics for long-term immunity of the conjugate vaccines [8,9,10]. Additionally, immunization schedules were being adapted to the changing epidemiology of meningococcal diseases as a result of direct and indirect (herd) effects conferred by routine immunization [2,11,12,13,14]. In this study, we describe the epidemiology of the invasive meningococcal disease in Spain, in the context of the frequent changes in immunization schedules together with the wide implementation of meningococcal B and ACWY vaccines through the private market. Our study highlights the age-stratified risk of the disease, which is a crucial consideration in the decision-making of parents, clinicians, and immunization policymakers for the use of meningococcal vaccines. These data could also provide a framework for other countries to adapt their respective meningococcal vaccination policies in response to the dynamic epidemiology of the meningococcal disease. 

## 2. Materials and Methods

### 2.1. Study Design

This was a retrospective review of a national hospital registry in Spain. This was an ecological study describing the hospitalization trend across the different periods of implementation of the routine meningococcal vaccines. 

### 2.2. Data source

The Spanish National Health System (Sistema Nacional de Salud, SNS) manages all the public healthcare services, including hospitals in Spain. All hospitals are required to submit their respective basic clinical dataset to the Conjunto Minimo Basico Datos (CMBD), which includes but is not limited to age, sex, diagnosis, length of stay, and outcome. All health facilities in the country submit hospital admissions and outpatient clinic visits to the registry. This system uses clinical codes from the *International Classification of Diseases, Ninth Revision, Clinical Modification* (ICD-9-CM; Spanish version: *Modificación Clínica Clasificación Internacional de Enfermedades*, CIE-9-MC) from 1998 to 2015 and later shifted to *International Classification of Diseases, Tenth Revision, Clinical Modification* (ICD-10-CM; Spanish version: *Modificación Clínica Clasificación Internacional de Enfermedades*, CIE-10-MC) from 2016 to 2017. This dataset has been used by the Spanish Health Ministry for their decision-making [15] and has been a resource for many publications [15,16]. Detailed characteristics of the database were described in a separate publication [16]. 

### 2.3. Case Classification

We defined invasive meningococcal disease as any physician-diagnosed case of *Neisseria meningitides* infection, such as, but not limited to, sepsis and meningitis. Meningococcal disease is a mandatory notifiable disease in Spain. All presumed cases are tested, and confirmed cases have microbiological confirmation using antigen testing, culture, or nucleic acid test from a normally sterile site. 

Because we used a national hospital registry, we obtained our confirmed cases using ICD-9-CM diagnosis, as shown in Box 1. In cases with multiple meningococcal diagnoses, the first entry was used as the primary diagnosis. We obtained cases registered from 1 January 1998 to 31 December 2017.

Box 1 **Case definition by hospital diagnosis.**

*International
Classification of Diseases, Tenth Revision, Clinical Modification (ICD-10-CM;
Spanish version: Modificación Clínica Clasificación Internacional de
Enfermedades, CIE-10-MC)* from 2016 to 2017A39
Infección meningocócica (meningococcal infection) A39.0
Meningitis meningocócica (meningococcal meningitis) A39.2
Meningococemia aguda (acute meningococcemia) A39.3
Meningococemia crónica (chronic meningococcemia) A39.4
Meningococemia, no especificada (meningococcemia, non-specific) A39.5
Enfermedad cardiaca debida a meningococo (heart disease due to meningococcus) A39.51
Endocarditis meningocócica (meningococcal endocarditis)*International
Classification of Diseases, Ninth Revision, Clinical Modification (ICD-9-CM;
Spanish version: Modificación Clínica Clasificación Internacional de Enfermedades,
CIE-9-MC)* from
1998 to 2015036-Infeccion
Meningococica (meningococcal infection) 036.0-Meningitis
Meningococica (meningococcal meningitis) 036.1-Encefalitis
Meningococica (meningococcal encephalitis) 036.2-Meningococemia
(meningococcemia) 036.3-Sindrome
De Waterhouse-Friderichsen, Meningococico (Waterhouse–Friderichsen Syndrome,
meningococcal) 036.4-Carditis
Meningococica (meningococcal carditis) 036.40-Carditis
Meningococica Sin Especificar (meningococcal carditis, unspecified) 036.41-Pericarditis
Meningococica (meningococcal pericarditis) 036.42-Endocarditis
Meningococica (meningococcal endocarditis) 036.43-Miocarditis
Meningococica (meningococcal myocarditis) 036.8-Otras
Infecciones Meningococicas Especificadas (other specified meningococcal
infections) 036.81-Neuritis
Optica Meningococica (meningococcal neuritis optica) 036.82-Artropatia
Meningococica (meningococcal arthropathy)

### 2.4. Data Analysis

Data were tabulated as counts and represented as percentages as applicable. The data were disaggregated into the year of admission and further into age groups (<1 year, 1–14 years, 15–64 years, >64 years old). Trends of hospitalization were expressed in a graph across time. We computed for the average number of hospitalizations per year and annual incidence of hospital admissions (per 100,000 persons, hospitalization rate) across different periods of routine meningococcal immunization—namely, (a) pre-routine immunization (1998–1999), (b) routine immunization with MenC vaccine using three primary series (2001–2004), (c) routine immunization with MenC vaccine using 2 primary and 1 booster dose (2006–2012), and (d) routine immunization with MenC with the inclusion of adolescent booster dose at 12 years old (2014–2017). For the denominator, we used the age-specific population from the census of Spain in the respective years (*Instituto Nacional de Estadística*, https://www.ine.es accessed on 13 December 2019). 

Focusing on the pediatric age group, age-risk estimation was calculated using the highest incidence age group (<1 year of age) as reference. We compared the risk across different ages by computing the odds ratio (OR), considering the different contingency tables for each age group. We performed all the analyses using R Software, Version 3.0.2. The significance level used was *p* < 0.05, carried out using two-tailed tests. 

### 2.5. Ethical Consideration

CMBD is a publicly available database containing an aggregate analysis of hospitalization without any patient identification. We adhered to the data privacy laws prevailing during the time of study conduct (Law 15/1999, adopted on 13 December 2013, on Biomedical Research regarding the protection of personal data and privacy).

## 3. Results

### 3.1. Meningococcal Disease Hospital Admissions in Spain from 1998 to 2017

**Figure 1:**
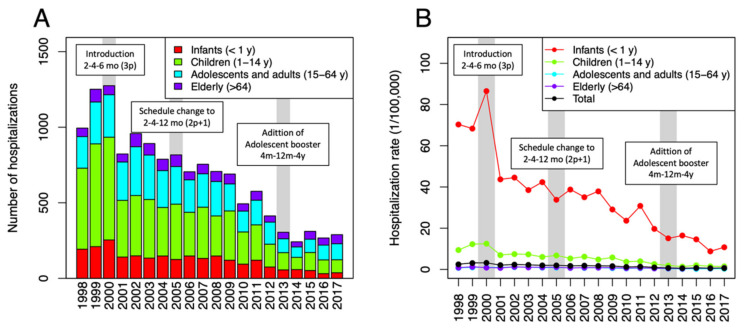
Trends on hospitalization of meningococcal diseases in Spain from 1998 to 2017: (**A**) number of hospitalizations, (**B**) hospitalization rate (expressed in 1/100,000 population).

In over 20 years of the study period, a total of 13,554 hospital admissions with meningococcal infections were identified through the Spanish national hospital registry (see Figure 1A). Overall, there was a downward trend in the hospital admissions with meningococcal disease in Spain. The burden of meningococcal disease gradually declined, comparing the pre-routine immunization period (1998–1999) with the post-routine immunization period (2001–2017), with average annual reported cases at 1123 cases/year to 590 cases/year, respectively, or a reduction of 47.5% from baseline (Table 1). Neurologic infections (meningitis and encephalitis) and sepsis comprised the most commonly reported meningococcal cases, with 7266 cases and 7510 cases, respectively. 

### 3.2. Hospital Admission of Pediatric Age Group

There were 2425 infants (<1 year) and 6053 children (1–14 years) hospitalized with meningococcal disease in Spain from 1998 to 2017. Cumulatively, the infant and child age group comprised the majority (62.5%) of all hospitalized meningococcal diseases in Spain—the majority of which were sepsis and meningitis, which constituted 97.7% among all the hospitalized meningococcal disease in the said pediatric age group (0–14 years). The annual average cases of invasive meningococcal diseases reported to CMBD during pre-routine immunization (1998–1999) in children was at 810 cases/year compared with 139 cases/year post-routine immunization period (2014–2017) for children 0–14 years old. Case reduction in infants was from 201 to 44 cases/year or a 78% decline from pre-routine immunization. In children (1–14 years), case reduction was 37.9%, from 608 to 377 cases/year. A higher reduction in proportion was seen in infants, but a higher reduction in the number of cases was seen in children.

### 3.3. Risk of Hospital Admission by Age

The incidence of hospitalization for meningococcal diseases decreased dramatically with age from 34.1 cases/100,000 in infants (<1 year) to 12.2 cases/100,000 in children 1–4 years of age. A similar pattern existed for both meningococcal meningitis and sepsis, such that infants had higher hospitalization compared with children (1–4 years). For meningococcal meningitis, the incidence was 18.3 cases/100,000 in infants (<1 year) compared with 6.6 cases/100,000 in children (1–4 years). For sepsis and meningococcemia, the incidence was 19.3 cases/100,000 for infants compared with 6.8 cases/100,000.

**Figure 2:**
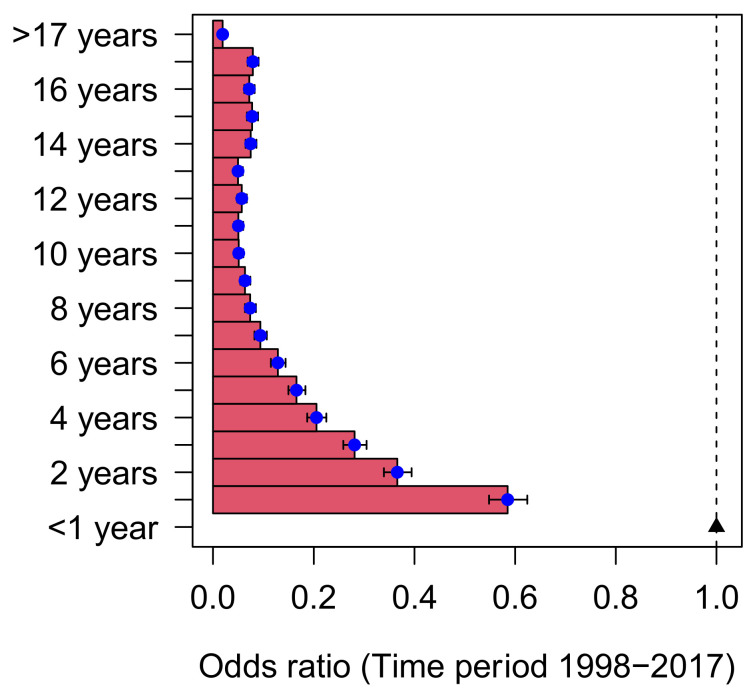
Odds ratio for hospitalization across the pediatric age group (infants <1 year used as reference).

Age-risk estimation was calculated for overall meningococcal disease, meningococcal meningitis, and meningococcal sepsis using the infants (<1 year) as the reference age group (Figure 2). There was a dramatic decline in risk from 1 year (OR 0.58 95%CI 0.53–0.63) to 4 years of age (OR 0.21 95% CI 0.18–0.24). The risk continued to decline to 13 years old (OR 0.05 95% CI 0.04–0.07). Afterward, a minimal upward trajectory at 14–17 years old (OR 0.08 95%CI 0.06–0.09) was seen. 

## 4. Discussion

In two decades of the observation period (1998–2017), we documented the dramatic decline in the total burden of meningococcal disease in Spain, at least partly due to routine vaccine use from late 2000 onwards. However, the highest burden of the disease was still disproportionately distributed among infants (<1 year old). The risk of hospitalization due to IMD decreased by 40% at 1 year of age and by 80% at 4 years of age.

Meningococcal C conjugate vaccines were introduced in Spain as a routine immunization for infants and as a campaign in older age group for selected autonomous regions beginning in the year 2000 [4,7]. Since then, the country has reached high immunization rates, with all regions achieving >95% coverage [17]. The vaccine impact was immediately seen after introduction, with a decline in hospitalization rate of invasive meningococcal disease by 58% (2.0 to 0.84/100,000 pop) and 68% (2.6 to 0.83/100,000 pop) on hospitalization rate for meningitis and sepsis, based on the literature [16,18]. The benefit was more prominently seen in children 0–14 years old. Across the years, the burden of illness has been declining, although cases are still disproportionately seen in the younger age groups. Thus in 2017, infants and children still comprise the majority, with >60% of the cases, and are continuously at risk for meningococcal diseases. Other studies in Spain, likewise, have documented the benefit of vaccine use in the country [5,6,19]. The most notable of which is the reduction in mortality and improvement in outcomes of meningococcal disease with the use of vaccines [18]. Past reports documented a declining disease incidence, as seen in the Spanish Disease Surveillance System [16,20,21]. Nasopharyngeal carriage of meningococcus has been studied in the country [22,23]. However, the carriage rates and circulating strains that are seen after serial changes in immunization policies are not yet known. Our study provides an estimation of the risk of hospitalization stratified into different ages, which is important information in vaccine decision-making. We also provided the burden of hospital admission that can be included in vaccine policy decisions for meningococcal vaccines.

The rate of hospitalization for meningococcus seen after the primary series of the vaccine could be explained by the waning immunogenicity of the vaccine. Prior studies demonstrated declining immunogenicity of the vaccine after a year of primary series [8,9]. The decreasing vaccine effectiveness was similarly seen in Spain, observed in adolescent age groups (14–17 years old) when routine immunization was introduced in infants [5,6]. These findings corroborate the seroprevalence study conducted in the Basque Country in Spain [24]. In 2009, a seroprevalence survey was carried out to determine the level of antibodies on MenC across the whole population. The study revealed that only 46.8% (children 2–5 years) and 36.1% (children 6–9 years) were seropositive, considering that the vaccines were routinely given in the area since 2000. The low seroprevalence was, likewise, seen 10 years after routine immunization in the UK [8]. As such, newer immunization schedules were explored to protect the high-risk population (direct effect) and to confer protection among other age groups (indirect or herd effect) [4,7,25].

With the persisting disproportionate distribution of cases in infants, the literature has suggested alternate immunization strategies on the use of meningococcal vaccines [26,27]. These alternate schedules exploit the capacity of the conjugate vaccines to confer herd effects. Routine immunization (including catch-up campaigns) with high coverage increases the herd immunity of the community, which in turn, expands the vaccine effects to other birth cohorts or with other infants with poor vaccine uptake. This phenomenon is usually mediated by the vaccine’s effect on eradicating the nasopharyngeal carriage in its recipient. Alternate immunization strategies include: (a) a three-dose infant series with or without catch-up immunization (for children <18 years of age), (b) a single-dose at 12 months old with or without catch-up immunization, or (c) a single-dose at 12 months with a booster dose at 12 years of age [27]. The optimal schedule is still a subject of debate among experts. The balance between direct protection through long-term immunity and herd effect through the eradication of nasopharyngeal carriage is a common dilemma for most policymakers and immunization managers. Thus, there are varying and opposing views on the manner of implementation, specifically at which target age groups the vaccine should be given [2,3,26]. Crucial factors among this decision are local circulating meningococcal strains, morbidity rate, case fatality rate, the nasopharyngeal carriage in the population, and type of vaccine used. As such, countries seem to have different approaches to routine immunization for meningococcal vaccines. 

In this paper, we provided a national estimate of hospitalized meningococcal diseases in Spain. However, our findings have crucial weaknesses, including all the limitations in ecological study design, which should be considered in interpreting our results [28,29]. An ecological study design is particularly useful in assessing the impact of the vaccine, as traditional vaccine effectiveness designs (i.e., case-control and cohort) prove to be more challenging to conduct because of the eradication of the disease from the vaccine herd effect. However, these study designs do not correct for any confounding, nor do they directly relate the exposure (vaccine uptake) to the outcome (disease). Furthermore, we also present some limitations that are unique to our analyses. First, the reliability of the CMBD depends on the quality of the discharge report and the clinical history, as well as the accuracy of coding for the diagnosis. CMBD is based on physician diagnosis, rather than a systematic surveillance method of confirmation supported by microbiologic evidence. Nevertheless, hospital-based reporting of cases through CMBD closely approximates the reports of the national (RENAVE-Spanish national disease surveillance) and regional (European CDC) reports and could be used to estimate the disease incidence. Second, the review of records only spanned until 2017. Meningococcal disease epidemiology is dynamic, and long-term sequelae and other clinically relevant outcomes are rarely captured by the current surveillance system. As such, CMBD and the national surveillance system in Spain could underestimate the vaccine effects and its impact [30]. Third, we have limited data on the serogroup and the specific strain circulating in Spain across different time points. This information is crucial, as several meningococcal vaccines are available for use, targeting different meningococcal serogroups. Finally, we have no data on nasopharyngeal carriage and data on meningococcal carriage across different age groups. Vaccines are known to change not only the disease epidemiology but also the carriage and disease transmission in the community, and as such, additional information in this regard is helpful. 

Surveillance on vaccine-preventable diseases is the cornerstone in monitoring the impact of the immunization program, especially in the case of invasive meningococcal diseases with highly dynamic disease epidemiology. Improved case reporting, serogroup identification, and molecular typing of isolates are needed to describe these epidemiologic shifts accurately. Nasopharyngeal carriage data across different age groups, including the impact of vaccination on the carriage rates, are also needed to provide additional evidence on the indirect effect of the vaccine. Likewise, immunization policies should also adapt to these epidemiologic changes. In 2015, surveillance data showed the predominance of B serogroup in the population, explaining at least half of the cases. Though a MenB vaccine was authorized for use in the country since 2014, its use was only recommended and reimbursed for high-risk populations [31], except for the Castilla-Leon Region, which started a routine MenB infant immunization. In the rest of the country, MenB vaccines can only be accessed through the private market but reached significant uptake through clinicians’ recommendations [32]. More recently, in March 2019, the government introduced the use of the Men-ACYW vaccine as a response to the increasing non-MenC serogroup in the community [33]. The vaccine is to be given in adolescents aged 12 years old and is expected to have a direct impact in the same age group until 17–18 years old when the disease transmission is also increased. As of the latest publication, MenB vaccines have no effect on nasopharyngeal carriage, which precludes any indirect effect (herd effect) in the population [34].

An appropriate schedule for a routine immunization program has to consider which age group is at risk of having the disease. Our analysis matches the surveillance data and the past seroprevalence study, which identifies infants and young children as still being at the highest risk for meningococcal disease. These findings, together with the growing evidence on long-term immunogenicity, impact on the nasopharyngeal carriage, and capacity for herd effect, should be considered for a more relevant immunization schedule.

## Figures and Tables

**Table 1:** Hospitalization rate of meningococcal infections disaggregated into different periods *.

Period	1998–1999	2001–2004	2006–2012	2014–2017
Vaccination	Pre-routine immunization	Routine immunization of MenC (3p) ^1^	Routine immunization of MenC (2p + 1) ^1^	Routine immunization of MenC^1^ (2p + 1 and adolescent booster)
Invasive meningococcal disease				
<1 year	69.34	42.31	30.74	12.64
1–14 years	191.25	106.05	61.84	25.26
15–64 years	0.9	1.02	0.67	0.33
>64 years	1.07	1.03	0.75	0.58
TOTAL	3.3	2.47	1.73	0.76
Meningococcal meningitis				
<1 year	35.66	21.9	16.7	6.9
1–14 years	102.24	55.48	32.24	14
15–64 years	0.5	0.57	0.35	0.19
>64 years	0.55	0.55	0.37	0.31
TOTAL	1.76	1.32	0.91	0.43
Meningococcal sepsis and meningococcemia				
<1 year	39.81	23.81	17.42	6.81
1–14 years	109.15	58.34	35.19	13.87
15–64 years	0.5	0.54	0.38	0.16
>64 years	0.66	0.57	0.43	0.32
TOTAL	1.88	1.35	0.98	0.41

* values expressed as hospital admission per 100,000 population. ^1^ Meningococcal serogroup C conjugate vaccine.

## Data Availability

Source data are managed by the Ministerio de Sanidad, Consumo y Bienestar Social, and are publicly available at http://pestadistico.inteligenciadegestion.msssi.es/publicoSNS/comun/ (accessed on 13 December 2019).
